# Prospective purification of perivascular presumptive mesenchymal stem cells from human adipose tissue: process optimization and cell population metrics across a large cohort of diverse demographics

**DOI:** 10.1186/s13287-016-0302-7

**Published:** 2016-03-30

**Authors:** C. C. West, W. R. Hardy, I. R. Murray, A. W. James, M. Corselli, S. Pang, C. Black, S. E. Lobo, K. Sukhija, P. Liang, V. Lagishetty, D. C. Hay, K. L. March, K. Ting, C. Soo, B. Péault

**Affiliations:** British Heart Foundation Centre for Vascular Regeneration & Medical Research Council Centre for Regenerative Medicine, University of Edinburgh, Edinburgh, UK; Department of Plastic and Reconstructive Surgery, St Johns Hospital, Howden Road West, Livingston, UK; Orthopaedic Hospital Department of Orthopaedic Surgery and the Orthopaedic Hospital Research Center, University of California, Los Angeles, CA USA; BD Biosciences, San Diego, CA USA; Bone and Joint Research Group, Institute of Developmental Sciences, University of Southampton, Southampton, UK; Department of Surgery, School of Veterinary Medicine and Animal Science, University of São Paulo, São Paulo, Brazil; Department of Emergency Medicine, Kaweah Delta Health Care District, Visalia, CA USA; Department of Urology, David Geffen School of Medicine, University of California at Los Angeles, Los Angeles, CA USA; Department of Pathology and Laboratory Medicine, David Geffen School of Medicine, University of California at Los Angeles, Los Angeles, CA USA; Indiana Center for Vascular Biology and Medicine, Krannert Institute of Cardiology, and Vascular and Cardiac Center for Adult Stem Cell Research, Indiana University, Bloomington, IN USA; Division of Growth and Development and Section of Orthodontics, School of Dentistry, University of California, Los Angeles, CA 90095 USA; Division of Plastic and Reconstructive Surgery, Department of Surgery and the Orthopaedic Hospital Research Center, University of California, Los Angeles, CA 90095 USA; Department of Orthopaedic Surgery and the Orthopaedic Hospital Research Center, University of California, Los Angeles, CA 90095 USA

**Keywords:** Mesenchymal stem cells, Adipose tissue, Adipose-derived stem cell, Cell sorting, Flow cytometry, Pericyte, Tunica adventitia

## Abstract

**Background:**

Adipose tissue is an attractive source of mesenchymal stem cells (MSC) as it is largely dispensable and readily accessible through minimally invasive procedures such as liposuction. Until recently MSC could only be isolated in a process involving *ex-vivo* culture and their *in-vivo* identity, location and frequency remained elusive. We have documented that pericytes (CD45-, CD146+, and CD34-) and adventitial cells (CD45-, CD146-, CD34+) (collectively termed perivascular stem cells or PSC) represent native ancestors of the MSC, and can be prospectively purified using fluorescence activated cell sorting (FACS). In this study we describe an optimized protocol that aims to deliver pure, viable and consistent yields of PSC from adipose tissue. We analysed the frequency of PSC within adipose tissue, and the effect of patient and procedure based variables on this yield.

**Methods:**

Within this twin centre study we analysed the adipose tissue of *n* = 131 donors using flow cytometry to determine the frequency of PSC and correlate this with demographic and processing data such as age, sex, BMI and cold storage time of the tissue.

**Results:**

The mean number of stromal vascular fraction (SVF) cells from 100 ml of lipoaspirate was 34.4 million. Within the SVF, mean cell viability was 83 %, with 31.6 % of cells being haematopoietic (CD45+). Adventitial cells and pericytes represented 33.0 % and 8 % of SVF cells respectively. Therefore, a 200 ml lipoaspirate would theoretically yield 23.2 million viable prospectively purified PSC - sufficient for many reconstructive and regenerative applications. Minimal changes were observed in respect to age, sex and BMI suggesting universal potential application.

**Conclusions:**

Adipose tissue contains two anatomically and phenotypically discreet populations of MSC precursors – adventitial cells and pericytes – together referred to as perivascular stem cells (PSC). More than 9 million PSC per 100 ml of lipoaspirate can be rapidly purified to homogeneity using flow cytometry in clinically relevant numbers potentially circumventing the need for purification and expansion by culture prior to clinical use. The number and viability of PSC are minimally affected by patient age, sex, BMI or the storage time of the tissue, but the quality and consistency of yield can be significantly influenced by procedure based variables.

**Electronic supplementary material:**

The online version of this article (doi:10.1186/s13287-016-0302-7) contains supplementary material, which is available to authorized users.

## Background

In a series of pioneering experiments in the 1960s, Alexander Friedenstein et al. [1] identified a population of cells from rodent bone marrow that adhered to culture vessels, formed colonies, could differentiate into osteoblasts in culture, and generated bone when implanted ectopically in vivo [2, 3]. Friedenstein termed these cells colony forming unit fibroblast (CFU-F), until Arnold Caplan [4], in the early 1990s, coined the term mesenchymal stem cells (MSC). Since their initial description, these cells have been the focus of much attention for their ability to differentiate into multiple mesodermal cell lineages, to modulate the immune system, and to stimulate regeneration through trophic support and the secretion of cytokines [5].

Despite the ultimate desire to translate MSC research into novel therapies, our understanding of these cells had been based principally on observations made in vitro on cells of undocumented purity and homogeneity, in ignorance of their anatomical location and physiological role in natural and pathological processes. MSC have been enlisted from bone marrow and multiple other tissues, according to their ability to adhere and grow on plastic [6]. This suggested that these cells possess a common identity and a widespread anatomical distribution but provided little insight into location, cellular phenotype, frequency, and specific properties of these cells. Some of these questions were answered when Crisan et al. [7] demonstrated that microvascular pericytes in multiple human fetal and adult tissues express MSC markers, and that when purified to homogeneity by fluorescence-activated cell sorting (FACS) and cultured they are identical to conventional MSCs in terms of morphology, phenotype, and function.

This led to the conclusion that pericytes, defined as CD31^−^CD45^−^CD34^−^CD146^+^, represent an origin of the MSC grown in culture, a finding that has been validated by other groups [8, 9]. Subsequently, a second population of anatomically and phenotypically distinct CD31^–^CD45^–^CD34^+^CD146^–^ cells with identical function to conventional MSCs has been identified that reside in the adventitial layer of larger blood vessels [10, 11]. Henceforth, we will refer to these two populations collectively as perivascular stem cells (PSC). Since their description, PSC have been confirmed to behave in vitro and in vivo like MSC, and to be equal in function, if not superior in some instances, to other stem and progenitor populations (reviewed in [12]).

Despite promising findings, there are challenges to address prior to the successful translation and wider clinical use of MSC. Because these cells are procured in low yield from tissues such as bone marrow, they are typically expanded and “purified” based on adherence to plastic under Good Manufacturing Practice compliant conditions—a costly, labor-intensive, and extended process requiring ex vivo culture prior to transplantation. The potential risks entailed by in vitro expansion include infection and immunogenicity due to the exposure of cultured cells to animal-based supplements [13]. Extended periods of in vitro expansion adversely impact the function of these cells, resulting in reductions in their chondrogenic, adipogenic, and osteogenic potentials [14–16]. Higher passage cells show modified and diminished expression of chemokine receptors and adhesion molecules resulting in lower response to chemokines and increased senescence [17]. Concerns have also been raised about the development of genetic instability and the potential for malignant transformation in cultured cells. In addition there are significantly more stringent regulatory hurdles that must be addressed with the use of cultured cells compared with those that have been minimally manipulated [18].

In an attempt to eliminate many of these issues, some groups have investigated the use of an adipose-derived stromal vascular fraction (SVF) as a source of MSC/progenitor cells that bypasses the need for in vitro culture and may be delivered at the point of use, requiring only basic preparation such as enzymatic digestion, washing, and centrifugation. Whilst the SVF may eliminate the requirement of ex vivo expansion, it is a very heterogeneous cell population containing endothelial cells, hematopoietic and inflammatory cells, and fibroblasts as well as cellular debris. This cellular heterogeneity may limit the regenerative potential of the SVF when compared with a more homogeneous MSC population, as has been demonstrated in models of osteogenesis [19].

It is therefore clear that there is a dichotomy in the current approaches to delivering MSC for clinical use. Methods relying on in vitro culture as a means of selection provide a relatively enriched but still undefined cell population and in addition incur the practical, financial, ethical, and regulatory problems this method manifests. Those methods which use the SVF do so at the expense of product identity, purity, and function. We have therefore sought to develop methods for the prospective purification of homogeneous populations of MSC based on our understanding of the exact anatomic location and identity of their native ancestors. Using multicolor FACS, we have purified cells from a range of human adult and fetal tissues [7, 12]. This early work was able to deliver distinct populations of cells that were subsequently expanded in vitro for further characterization, analysis, and experimental work. As our interest in these cells has developed and their potential for immediate clinical use was explored, we established that large numbers of the cells recovered immediately from FACS were of poor quality, and were in the process of dying. This had been previously overlooked in our in-vitro populations because only the healthy cells would adhere and expand. For PSC to be used immediately after FACS, we would need to demonstrate that it is possible to recover pure, viable, and consistent yields of cells. We therefore sought to refine our protocols to maximize not only total cell yield, but maximum viable cell yield, purity, and consistency leading to the development of an optimized protocol that we describe in this work. Using this optimized protocol, we document the cellular composition of human adipose tissue using flow cytometry across a wide demography of donors, and to establish whether PSC can be prospectively purified in clinically relevant numbers, circumventing the need for ex vivo expansion. Furthermore we examined the patient and procedure based variables that may influence this yield. Results were collected from 131 individual lipoaspirates processed in two distinct research centers by different investigators.

## Methods

This was a twin-center study based at the University of California at Los Angeles (UCLA), USA and the University of Edinburgh, UK. Adipose tissue was collected with prior written consent from patients undergoing cosmetic lipectomy procedures. Permission for the collection of tissue and subsequent research was granted in Edinburgh by the South East Scotland Research Ethics Committee (Reference: 10/S1103/45), and was unnecessary in Los Angeles because fat was collected under an institutional review board exemption, being considered medical waste.

Tissue was processed and flow cytometry performed using our previously published protocols [12] and the modifications detailed in this manuscript (Additional file 1). Briefly, lipoaspirate was washed in phosphate buffered saline and centrifuged to separate the fat from the oil and liquid phases. Fat was combined vol/vol with 125 CDU/ml type II collagenase (collagenase from *Clostridium histolyticum*, C6885; Sigma Aldrich, St Louis, Mo, USA) in Dulbecco’s modified Eagle’s medium + 3.5 % bovine serum albumin (BSA) (Cohn Fraction V A7906; Sigma) and digested for 45 minutes at 37 °C in a shaking water bath (200 rpm). Samples were centrifuged to isolate the SVF which was subsequently filtered through a 100 μm filter and then a 70 μm filter before red cells were lysed. Finally, the SVF was filtered through a 40 μm filter prior to manually counting live cells using trypan blue staining and a hemocytometer. The SVF was then stained with the following antibodies: CD31, CD34, and CD45 (all from BD Biosciences, San Jose, CA, USA) and CD146 (AbD Serotec, Raleigh, NC, USA). Flow cytometry was performed using a BD FacsAria II or III sorter fitted with a 100 μm nozzle following adequate compensation controls using either single stained cells or compensation beads. An initial forward scatter (FSC) vs side scatter (SSC) gate was used to identify cells, followed by gates to select for single cells. Cells not stained by 4′,6-diamidino-2-phenylindole (DAPI) were gated as viable, and hematopoietic and endothelial cells were eliminated based on CD45 and CD31 expression respectively. PSC were subsequently purified from the CD31^–^/CD45^–^ cell fraction according to their differential expression of CD34 and CD146 (pericytes: CD146^+^, CD34^–^, CD31^–^, CD45^–^; adventitial cells: CD146^–^, CD34+, CD31^–^, CD45^–^) [7, 10].

Flow cytometry data were analyzed using FlowJo (v. 10.0, FlowJo, Ashland, OR, USA) or Diva (v. 6.0, BD Biosciences, San Jose, CA, USA) software, and reviewed by CCW, IRM, WRH, and BP to ensure that accurate and standardized gating strategies were employed across sites and between users. Statistical analysis was performed using JMP10 software (SAS Institute Inc., Cary, NC, USA). Normally distributed nominal data were analyzed using a paired *t* test, while bivariate fit using regression analysis was used for continuous data. Multivariate analysis was performed using multiple linear least-squares regression. Data were considered significant when *p* <0.05.

## Results

### Demographic and PSC parameter analysis

Demographic information as well as cell yield, viability, and subpopulation sort statistics are summarized in Table 1 for 131 unique donor samples.

**Table 1:** Demographic data of the 131 donors

Demographic data (*n* = 131)	
Sex	Female = 112
	Male = 19
Age (years)	Mean = 41 (range 22–64)
BMI (kg/m^2^)	Mean = 26.5 (range 19–43)
SVF (cells × 10^6^)	Mean = 34.4 (range 4.7–120)
Viability (%)	Mean = 83 % (range 36–99)
Pericytes (%)	Mean = 8 % (range 0–55)
Adventitial cells (%)	Mean = 33.0 % (range 3–72)
PSC total (%)	Mean = 41 % (range 6–78)
PSC yield (cells × 10^6^)	Mean = 11.6 (range 1.1–47.2)

*BMI* body mass index, *PSC* perivascular stem cell, *SVF* stromal vascular fraction

### Analysis of SVF

**Fig. 1:**
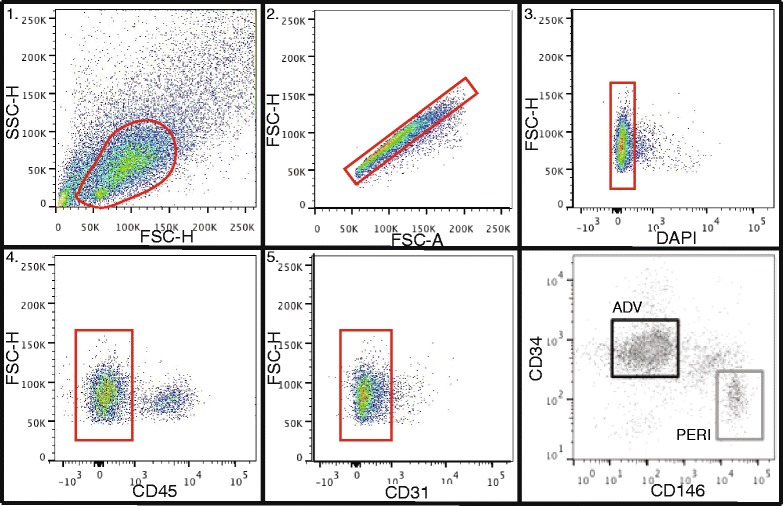
Gating strategies for the isolation of PSC from the SVF. Gate 1 = cells, Gate 2 = single cells, Gate 3 = live cells, Gate 4 = CD45^**−**^ (nonhaematopoietic cells), Gate 5 = CD31^–^ (nonendothelial cells), Gate ADV = adventitial cells (CD31^**−**^, CD45^**−**^, CD34^**+**^, CD146^**−**^), and Gate PERI = pericytes (CD31^**−**^, CD45^**−**^, CD34^**+**^, CD146^**−**^). *DAPI* 4',6-diamidino-2-phenylindole, *FSC* forward scatter, *SSC* side scatter

The SVF was isolated from total fat by collagenase digestion. The mean yield of nucleated cells was 34.4 × 10^6^ per 100 ml of lipoaspirate (median: 30.0 × 10^6^; standard deviation (SD): ± 21.0 × 10^6^; range: 4.7 × 10^6^–120 × 10^6^; *n* = 131). Upon FACS analysis, an initial FSC vs SSC gate was set to delimit PSC that occupy a characteristic region of the cytogram in terms of size and internal complexity amongst the diverse mixture of cells that comprise the SVF. Significantly, this demonstrated that the majority of the SVF in many samples is comprised of dead and dying cells and cellular debris. Gating to select single cells is necessary to prevent the collection of non-PSC due to incomplete collagenase digestion and/or cell aggregation post isolation and during the processing and staining of the cells prior to sorting. Since collagenase dissociated cells approximate a sphere, the height-to-width ratio should maintain a constant value—or said differently, the height and width of the cells should scale proportionally to the area. Single cell selection by FACS is possible using the gating parameters shown in Fig. 1. Viable cells possess an intact plasma membrane and exclude DAPI, allowing cell viability to be assessed (mean cell viability: 83 %; median: 84 %; SD: ±12 %; range: 36–99 %; *n* = 131). CD45^**+**^ haematopoietic cells and CD31^**+**^ endothelial cells averaged 34 % (median: 33 %; SD: ±16 %; range: 1–73 %; *n* = 113) and 4 % (median: 3 %; SD: ±4 %; range: 0.1–16 %; *n* = 17), respectively, of total live cells in the SVF. The mean proportion of PSCs was 41 % (median 42 %; SD: ±16 %; range: 6–78 %; *n* = 124) with a mean proportion of pericytes of 8 % (median 5 %; SD: 8 %; range: 0–55 %; *n* = 131), and adventitial cells of 33 % (median 34 %; SD: ±16 %; range: 3–72 %; *n* = 131). This represents a mean yield of 11.6 × 10^6^ PSCs (median 10.0 × 10^6^; SD: 8.6 × 10^6^; range: 1.1 × 10^6^–47.2 × 10^6^; *n* = 124) per 100 ml of lipoaspirate.

### Effects of demographics on cell yield

**Fig. 2:**
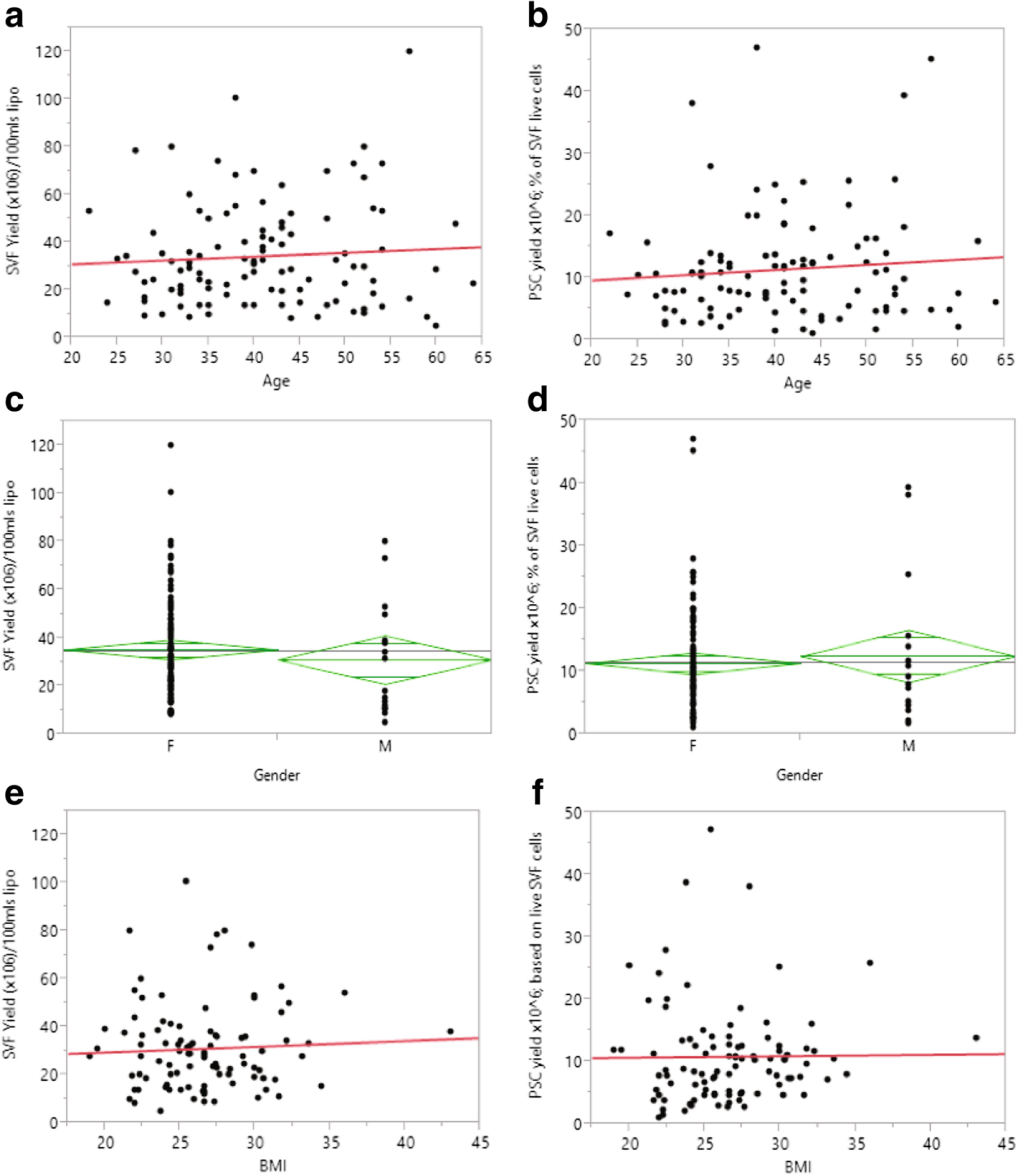
**a, b.** Linear fit of SVF yield and PSC yield (× 10^6^) per 100 ml of lipoaspirate with respect to donor age (*n* = 124, *R* = 0.07 and 0.09 respectively). **c, d.** One-way analysis of variance of SVF and PSC yields (× 10^6^) vs donor gender (*n* = 131; male = 19, female = 112): *green diamonds* reflect the mean yield (center line) and 95 % confidence interval (vertical span) for each gender, and the grand mean in *gray*. Linear fit of SVF yield **e** and proportion of PSCs comprising the SVF **f** with respect to donor BMI (*n* = 97, *R* = 0.05 and 0.01 respectively). *BMI* body mass index, *F* female, *lipo* lipoaspirate, *M* male, *PSC* perivascular stem cell, *SVF* stromal vascular fraction (Color figure online)

The mean age of donors was 41 years (range: 22–64, *n* = 124 in Table 1). There were no differences observed in either the total number of viable SVF cells or the proportion of PSC as a percentage of live cells with age, with linear correlation coefficients (*R*) of 0.07 and 0.09, respectively (Fig. 2a, b). No statistical difference was observed in the yield of SVF cells (*p* = 0.34) or viable PSC (*p* = 0.79) between genders (Fig. 2c, 2d, respectively). Although the proportion of PSC (as a percentage of live cells) was significantly higher in male vs. female donors, with mean PSC proportions of 47 % vs 40 %, respectively (*p* = 0.05, one-tailed *t* test), the average SVF yield (male: 30 × 10^6^ cells vs female: 35 × 10^6^ cells) and cell viabilities (male: 82 % vs female: 83 %) were correspondingly lower, although not significantly, in males, resulting in a zero sum scenario (data not shown). Body mass index (BMI) had no significant effect on either the total yield of SVF cells or the proportion of PSC as a percentage of live cells (*R* = 0.05 and 0.01, respectively; Fig. 2e, f). Multivariate analysis was performed using multiple linear least-squares regression for all demographic data and cold storage times, resulting in *F* ratio = 2.99, *p* = 0.035. The only factor within these parameters that demonstrated any significant correlation to cell yield was the cold storage time (*t* ratio = 2.99, *p* = 0.004), which is discussed further below.

### Effect of cold storage time on cell yield

**Fig. 3:**
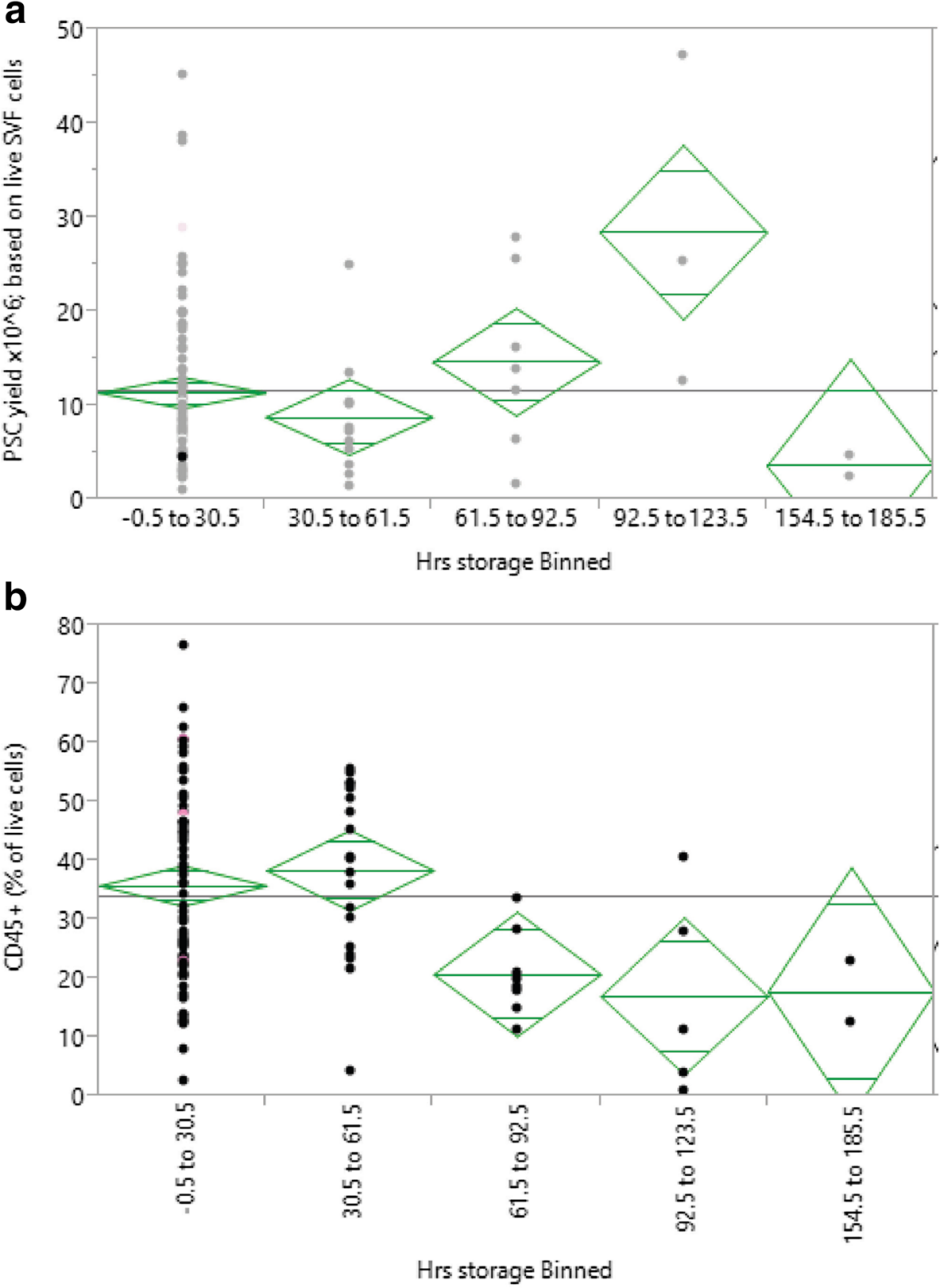
One-way analysis of variance analysis of the number and proportion of cells recovered with respect to storage time at 4 °C. **a** PSC yield (× 10^6^) per 100 ml of lipoaspirate showing a gradual increase with longer storage times; however, this is a relative effect due to the deceasing number of CD45^+^ hematopoietic cells seen in **b**. *Green diamonds* indicate the mean and 95 % confidence interval for each storage time interval, while the *gray line* represents the grand or overall mean. *Hrs* hours, *PSC* perivascular stem cell, *SVF* stromal vascular fraction (Color figure online)

After surgical removal, adipose tissue was stored at 4 °C until analyzed. The majority of samples were processed within 24 hours following surgery; however, some samples were stored for up to 7 days. When split into discreet time points and analyzed using the Tukey–Kramer (honest significant difference) test, we observed a general increase in the proportion of PSC recovered from the SVF with increasing time (not significant) (Fig. 3a); however, the absolute numbers remained consistent and the relative rise was in fact due to a reduction in the proportion of CD45^+^ haematopoietic cells (Fig. 3b).

### Statistical process control over the prospective isolation of PSCs

**Fig. 4:**
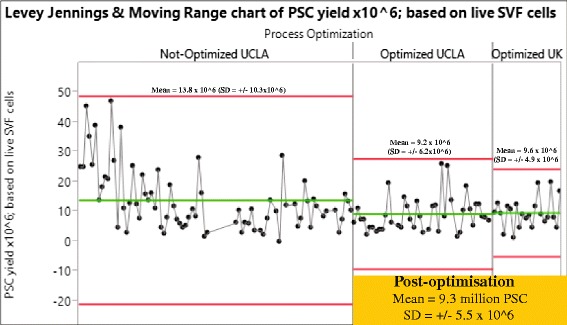
Statistical control chart demonstrating that optimization improved the reproducibility of PSC isolation and confirming that both the UCLA and UK isolation processes are under statistical control. A Levey-Jennings chart depicting individual data points for PSC yield (×10^6^) obtained from 100 ml of lipoaspirate using the UCLA isolation process, before and after optimization, as compared with the UK process (*n* = 131). The central *green line* represents the general mean and is delimited by upper and lower control limits (*red lines*) based upon a 3σ interval. *PSC* perivascular stem cell, *SD* standard deviation, *SVF* stromal vascular fraction, *UCLA* University of California at Los Angeles (Color figure online)

**Fig. 5:**
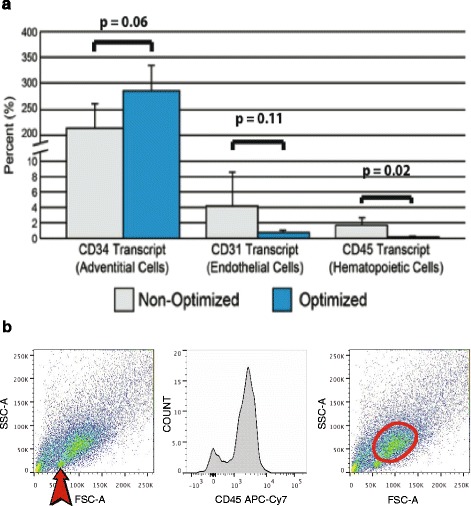
Improvements in PSC purity. **a** Optimization of the PSC isolation process has led to increased purity of cells, as indicated by enrichment for the adventitial cell antigen CD34 (*left*), and the dramatic reduction in the endothelial cell antigen CD31 (*middle*) and haematopoietic marker CD45 (*right*) by real-time quantitative PCR (*n* = 7; pre optimization = 4, post optimization = 3). **b** (*Left*) FSC vs SSC demonstrating the population of lymphocytes (*arrow*). (*Centre*) Confirmation of lymphocytes by demonstration of CD45^+^ phenotype of the subpopulation. (*Right*) Selection of the CD45^–^ depleted cellular fraction for subsequent analysis. *FSC* Forward scatter, *PSC* perivascular stem cell, *SSC* side scatter

Since 2013, both groups at UCLA and Edinburgh have been using the same protocol based on the improvements and developments made during the process of optimization. To assess the extent to which our process was consistent, reproducible, and under statistical control we used Levey-Jennings charts that show control limits 3 SDs above (upper control limit) and below (lower control limit) the process mean. When PSC yields (per 100 ml of lipoaspirate) obtained using the optimized protocol were compared with the non optimized earlier protocols, it was apparent that our optimized protocol had resulted in improvements in the reproducibility and purity of PSC isolation (Figs 4 and 5). Levey-Jennings charts depicting individual data points (Fig. 4) showed that mean PSC yields were reduced following process optimization (from 13.8 to 9.3 million PSC per 100 ml of lipoaspirate) but resulted in quite similar PSC yields between the UCLA and Edinburgh groups (9.2 and 9.6 million PSC)—it should be noted that the viability of cells post optimization was higher. Furthermore, upper and lower control limits were greatly improved following process optimization, resulting in a 53 % reduction in sample SD: 5.5 × 10^6^ vs 10.3 × 10^6^ PSC for the optimized and nonoptimized process, respectively. The statistical control charts thus demonstrate that our current protocol has improved the reproducibility of PSC isolation and confirm that both the UCLA and Edinburgh isolation processes are under statistical control. At the same time, contamination of sorted PSC by CD45^**+**^ haematopoietic cells and CD31^**+**^ endothelial cells has decreased, yielding greater than 99.5 % depletion of these cells based upon the detection of protein tyrosine phosphatase, receptor type C (CD45) and platelet endothelial cell adhesion molecule PECAM1 (CD31) transcripts by real time quantitative PCR (Fig. 5).

## Discussion

Regenerative medicine is defined as the process of replacing, engineering, or regenerating cells, tissues, or organs to restore or establish normal function. Paramount to the clinical translation of basic research into adult stem cell therapies is the identification of cell sources and methods that minimize any potential risks to the patient. These risks are mitigated when pure, well-defined populations of cells are used that have not undergone the extensive ex vivo manipulation that culturing entails with its associated risks due to infection, immunogenicity, and genetic instability. Additionally, the future clinical adoption of MSC for regenerative therapies requires a precise delineation of cellular identity, purity, and potency as specified by the Food and Drug Administration in the April 2008 Content and Review of Chemistry, Manufacturing, and Control Information for Human Somatic Cell Therapy Investigational New Drug Applications [20], and as reinforced in the November 2013 Guidance for Industry: Preclinical Assessment of Investigational Cellular and Gene Therapy Products [21]. Clearly, progress toward the clinical use of MSC awaits a standardized manufacturing process that will consistently produce sufficient numbers of MSC of defined identity, purity, and potency safely and from easily dispensable human tissues uncompromised by conditions that might alter their sterility, stability, immunogenicity, and genomic integrity.

Adipose tissue is an abundant source of perivascular associated regenerative cells that can be harvested in large quantities even from individuals of normal BMI with minimal morbidity. These PSC consist of a bipartite population of adventitial cells and pericytes which together embody the entire regenerative potential of adipose tissue and are well defined in their anatomical origin, immunophenotype, and contribution to fat tissue composition [7, 10–12, 14, 19, 22, 23]. Notwithstanding their tissue origin, purified PSC are indistinguishable from conventional MSCs in their multidifferentiation potential [7, 10–12, 14, 19, 24–29], and in vitro and in small animal studies have been shown to function in hematopoietic stem cell support [30], fibrosis [31–33], and bone [14, 19], muscle [34], pulmonary [35], peripheral nerve [36], white adipocyte [24], follicular dendritic cell [37], and cardiovascular [38–42] regeneration. In some of these models, the function of PSCs was equivalent or superior to that of conventional MSCs or other progenitor populations (reviewed in [12]). Furthermore, unlike conventional MSC, PSC do not require culture for isolation and or purification, and have been convincingly shown to induce greater bone formation than their unpurified counterpart cell populations in the SVF [19, 43]. In this present study, we have optimized a process to rapidly and consistently purify healthy and viable PSC from moderate amounts of lipoaspirate to high homogeneity by FACS [10, 12, 19, 44] in quantities theoretically sufficient to address many clinical needs.

Currently, PSC isolation relies on sophisticated FACS flow cytometers that are uncommon in clinical settings and costly to acquire, operate, and maintain. Although clinical-grade cell sorters are available [45], the widespread adoption of PSC and other sorted cell therapies will require the codevelopment of stem cell treatment centers like the recently funded California Alpha-stem Cell Clinics, which are technically staffed and equipped with such instruments [46].

This analysis of 131 samples represents the largest and most comprehensive analysis of adipose tissue stem cells to date. We demonstrate a theoretical yield of 11.6 million PSC per 100 ml of lipoaspirate; however, an average yield of 9.3 million PSC per 100 ml is a more realistic and consistent estimate of PSC based on process optimization and data compiled from two different laboratories at UCLA (9.2 million PSC from *n* = 109 samples) and Edinburgh (9.6 million PSC from *n* = 22 samples). Given that cosmetic liposuction procedures often exceed volumes of 1 l, more than sufficient cells could be prospectively isolated to satisfy a range of regenerative applications (Table 2) based on estimates obtained from clinical trials data (www.clinicaltrials.gov) and our own group. Nevertheless, *e*x vivo culture would still be necessary for systemic conditions and where multiple doses are required, such as graft versus host disease.

**Table 2:** Potential clinical applications and the amount of adipose tissue required to provide sufficient numbers of prospectively purified perivascular stem cells to eliminate the need for ex-vivo expansion

Clinical use	Estimated number of cells required	Amount of fat needed
Tissue engineered tendon	1.5 million/cm	12 ml/cm
Tissue engineered mandible	10 million/cm	80 ml/cm
Cartilage for nasal reconstruction	20 million	160 ml
Scaphoid nonunion	25 million	200 ml
Tibial nonunion^a^	40 million	320 ml
Total ear reconstruction	50 million	400 ml
Critical limb ischemia^a^	2 million cells/kg	16 ml/kg
Graft versus host disease^a^	0.5–13 million cells/kg	4.8–125 ml/kg
		(repeat doses required)

^a^Data from clinicaltrials.gov

The protocol presented in this work is based on the one originally developed to purify PSC from a variety of fetal and adult tissues [7], which was subsequently modified specifically for adipose tissue [19]. Through examining the health, quality, and purity of these PSC populations immediately after FACS, we noted that large numbers of PSCs were of poor quality and in the process of dying. We therefore sought to refine and optimize our protocol with emphasis on consistently delivering maximum numbers of pure and viable PSC.

There are a number of modifications that have been made to this current protocol when compared with that previously published by James et al. [19]. The most significant change made was to reduce the enzymatic digestion time down from 70 minutes to 45 minutes On close examination of the cells following digestion, we noted that although the length of digestion did not seem to have a major effect on total SVF yield (45 minutes = 34.4 × 10^6^ vs 70 minutes = 39.4 × 10^6^ nucleated cells/100 ml of lipoaspirate), the health of those cells recovered was adversely affected by longer digestion times—such that significant proportions of cells recovered immediately following FACS were dead or apoptotic. Two further filtration steps (100 and 40 μm) were introduced immediately following enzymatic digestion to remove debris that were more apparent with the shorter digestion time. When comparing the number and type of cells recovered between the current protocol and that published previously [19] it appears that there has been a large reduction in the percentage of pericytes retrieved (8 % vs 19.5 %), and an increase in the adventitial cells (33 % vs 23.8 %). It is our experience that this change is more a reflection on the number of healthy cells that can be recovered, and although the total proportion of PSC remains similar (41 % vs 43.2 %), the current protocol delivers healthier cells that are therefore more suitable for immediate use. We have noted that the pericyte population is especially prone to damage due to the longer digestion. Further changes were made through the addition of an endothelial cell specific antibody (CD31) to eliminate these cells as part of the FACS purification process, resulting in greater purity of the samples (Fig. 5a). This protocol relies on the selection of pericytes being CD146^+^ and adventitial cells being CD34^+^ but both of these groups were probably contaminated with endothelial cells because subsets express both CD34 and CD146. These cells were not present in subsequent analysis of in-vitro populations due to the unfavorable culture conditions for endothelial cells. We have also noted reductions in other contaminating populations such as the CD45^+^ hematopoietic cells (Fig. 5). Both protocols utilize a CD45 antibody to deplete these cells; however, we believe that as our experience of this process has improved we have also been able to set tighter FACS gates to eliminate these populations with more accuracy (Fig. 5b). In addition to the importance of being able to deliver a pure and defined clinical product, it has been shown that contamination of MSC by endothelial cells also inhibits their function [47, 48].

The ultimate goal is to translate our work to novel therapies that address a wide range of clinical needs. The protocol described here utilizes research-grade reagents and therefore consideration will need to be given to translating the work for clinical use. Wherever possible we have sought to utilize reagents that can also be bought at clinical grade; however, there are some reagents where alternatives may need to be sought, such as substituting human serum albumin for BSA. Further optimization will probably be required for this process, but the current work will act as a benchmark for subsequent studies.

One part of the process over which we had no control was the liposuction methods used by the surgeons recovering the adipose tissue. Whilst we have not specifically investigated the effect of different liposuction procedures on PSC yield, it is likely that variations in the type of liposuction cannula and hence the size of fat within the lipoaspirate will influence the digestion process and recovery of PSC. In all of the samples we processed, the adipose tissue was the by-product of a cosmetic procedure and so the type of liposuction performed was at the discretion of the surgeon. In future, if liposuction is to be performed with the primary intention of recovering cells for clinical use we would suggest that the liposuction procedure should also be examined, optimized, and standardized.

There are a number of limitations of this current study that need to be addressed. Whilst this study represents the single largest analysis of SVF and stem/progenitor cell content, the demographics of this cohort reflect the unique type of patient undergoing cosmetic plastic surgery, and might not reflect the full demographics of people requiring stem cell therapies. Generally patients undergoing cosmetic surgery are young to middle-age women and free from any significant comorbidities. Whilst the results suggest that age is not a factor in PSC yield, the oldest patient in our study was aged 64. Further studies are therefore required on patients at later ages. Furthermore, our study was limited to looking only at the number and viability of cells and did not examine their function or potency for particular regenerative purposes; however, the developmental potential of fat-derived PSC has been addressed in many other publications [14, 30, 49] and shown not to be significantly affected by gender, age, and BMI [19]. Although we have qualitative evidence to demonstrate that pericytes and adventitial cells have a similar function and potency [7, 10], detailed assays examining this have not been performed. This is an important issue that needs to be addressed because there is evidence from our group and others that specific subsets of cells within the MSC/PSC family may have specific and unique functions [30, 50].

By selecting a purified population of stem/progenitor cells, we are more likely to increase the efficacy of these cells by eliminating contaminating cells; however, the effects of patient lifestyle, genetic background, and other variables on the function of the resulting populations should be examined because both age and disease have been implicated in reduced function of conventional MSC [51]. This is particularly relevant if an allogeneic source of MSCs is to be proposed and defended as a viable alternative to autologous cells.

## Conclusions

Adipose tissue contains two anatomically and phenotypically discreet populations of MSC precursors—adventitial cells and pericytes—together referred to as PSC. More than 9 million PSCs per 100 ml of lipoaspirate can be rapidly purified to homogeneity using flow cytometry in clinically relevant numbers, potentially circumventing the need for purification and expansion by culture prior to clinical use. Modern high-speed flow cytometers can theoretically process cells at rates well in excess of 20,000 cells/second, thus meaning tissue could be processed and purified PSC returned and administered for therapeutic use within a single operative procedure. The number and viability of these cells are minimally affected by factors such as age, sex, BMI, and storage time in this cohort; however, further studies are required to examine the effects of age and pathology on the number and efficacy of these cells.

## Additional file

Additional file 1: presents the protocol and reagents for the purification of PSCs from adipose tissue. (DOC 50 kb)

## Abbreviations

BMI: Body mass index
BSA: Bovine serum albumin
CD: Cluster of differentiation
DAPI: 4′,6-Diamidino-2-phenylindole
FACS: Fluorescence-activated cell sorting
FSC: Forward scatter
MSC: Mesenchymal stem cell
PSC: Perivascular stem cell
SD: Standard deviation
SSC: Side scatter
SVF: Stromal vascular fraction
UCLA: University of California at Los Angeles
