# Supplementary material for: Prospective purification of perivascular presumptive mesenchymal stem cells from human adipose tissue: process optimization and cell population metrics across a large cohort of diverse demographics
Source: Stem Cell Res Ther. 2016 Mar 30;7:47. doi: 10.1186/s13287-016-0302-7 (PMC4815276; doi:10.1186/s13287-016-0302-7)
Supplement: Additional file 1: — presents the protocol and reagents for the purification of PSCs from adipose tissue. (DOC 50 kb) [file 13287_2016_302_MOESM1_ESM.doc]

**Protocol and reagents for the purification of PSC from adipose tissue**

1. **Preparation of SVF by enzymatic digestion of lipoaspirate**

1. Transfer 50mL of lipoaspirate into each of two 50mL falcon tubes.

2. Centrifuge the tubes at 1800 rpm for 10 min. After spinning, the tissue of interest will be between blood at the bottom and some liquid fat at the top of the tube.

3. Transfer the middle layer to a new 50mL tube with a 1mL tip.

4. With approximately 25mL of tissue/ tube, add 25mL of PBS to each and shake well.

5. Centrifuge the tubes again at 1800 rpm for 10 min.

6. Transfer the fat layer to new tubes.

7. Add 25mL of digestion solution to each tube and shake well. Transfer the tubes to the shaking water bath at 37°C 250rpm for 45 minutes.

8. After incubation, centrifuge the tubes at 1800 rpm for 10 min.

9. Aspirate the supernatant (containing oily fat and adipocytes) and resuspend each pellet in 25 ml of PBS 5mM EDTA. Disrupt the clumps as much as possible by pipetting up and down. Discard any persistent clumps.

10. Filter the tissue suspension through 100μm and 70μm cell strainers.

11. Top up the filtered solution with PBS-EDTA and centrifuge at 1500rpm for 10 min.

12. Aspirate/discard the supernatant and resuspend the pellet(s) in 10mL of RBC lysis buffer. Incubate at room temperature for 5-10min.

13. Add 20mL of PBS-EDTA to the tube(s) and filter the resulting suspension through a 40μm cell strainer.

1. As in Step 8, top up the filtered solution with PBS-EDTA and centrifuge at

1500 rpm for 10 min.

1. Aspirate/discard the supernatant and resuspend the pellet (comprised of the SVF) in 5-10mL PBS-EDTA to count.

16. Resuspend cells in 1000μL PBS for immunostaining.

Digestion solution (DMEM, Collagenase II 1mg/ml, BSA 3.5%)

Red Blood Cells Lysis buffer

Stock 1 8.3g ammonium chloride in 1000 ml water

Stock 2 20.59g Tris base in 1000 ml water after adjusting pH to 7.65 with 1M hydrochloric acid

9 parts of stock 1 are added to 1 part of stock 2 and the pH is adjusted pH to 7.65 with 1M hydrochloric acid

1. **Purification of PSC from SVF using FACS**
2. See figure 1 in main text for detailed description of gating strategies.
3. **Reagents / consumables**

| **Item** | **Supplier** | **Cat number** |
| --- | --- | --- |
|  |  |  |
| **Digestion** |  |  |
| DMEM + Glutamax | Gibco | 61965-026 |
| PBS | Sigma | D8537 |
| TypeII collagenase | Sigma | C6885 |
| BSA | Sigma | A7906 |
| FBS | Gibco | 10270-106 |
| Sterile cell strainers | Fisherbrand | 22363547 |
| 50ml Centrifuge tubes | Corning | 430828 |
|  |  |  |
| **FACS** |  |  |
| FACS tubes | Scientific Lab Supplies | 352054 |
| DAPI | Invitrogen | D1306 |
| Compensation beads | BD Biosciences | 552843 |
| CD146 A647 | AbD Serotec | MCA928A647 |
| CD31 PE | BD Biosciences | 555446 |
| CD34 FITC | BD Biosciences | 555821 |
| CD45 APC-Cy-7 | BD Biosciences | 557748 |
